# Causal relationships between plasma lipidome and diabetic neuropathy: a Mendelian randomization study

**DOI:** 10.3389/fendo.2024.1398691

**Published:** 2025-01-15

**Authors:** Zhaoxiang Wang, Zhong Liu, Qichao Yang, Huibo Qiao, Yong Yin, Zhiyong Zhao, Xuejing Shao

**Affiliations:** ^1^ Department of Endocrinology, First People’s Hospital of Kunshan, Kunshan, Jiangsu, China; ^2^ Department of Emergency Medicine, First People’s Hospital of Kunshan, Kunshan, Jiangsu, China; ^3^ Department of Endocrinology, Affiliated Wujin Hospital of Jiangsu University, Changzhou, Jiangsu, China; ^4^ Department of Endocrinology, Wujin Clinical College of Xuzhou Medical University, Changzhou, Jiangsu, China

**Keywords:** lipids, diabetic neuropathy, GWAS, Mendelian randomization analysis, causal relationship

## Abstract

**Background:**

Dyslipidemia is closely related to diabetic neuropathy. This study examined the potential causal relationship involving 179 lipid species and the disease.

**Methods:**

The pooled data on 179 lipid species and diabetic neuropathy were obtained from previous genome-wide association studies (GWAS). A Mendelian Randomization (MR) method was employed to investigate the potential causal link, and the robustness of the findings was confirmed through comprehensive sensitivity analyses.

**Results:**

Genetically, phosphatidylcholine might be associated with the risk of diabetic neuropathy. Upon adjusting for multiple comparisons, higher levels of phosphatidylcholine (16:0_20:2) (OR = 0.82, 95%CI: 0.73-0.91; *P* < 0.001, FDR = 0.033) and phosphatidylcholine (16:1_18:1) (OR = 0.77, 95%CI: 0.67-0.88; *P* < 0.001, FDR = 0.019) are associated with a decreased risk of diabetic neuropathy. Further multivariable MR (MVMR) analysis demonstrated the effect of genetically predicted phosphatidylcholine (16:1_18:1) remained after adjusting for body mass index (BMI) and glycated hemoglobin (HbA1c). Sensitivity assessments have confirmed the robustness of these findings, revealing no evidence of heterogeneity or pleiotropy.

**Conclusion:**

Our research linked certain lipid species with diabetic neuropathy risk, suggesting that targeting lipids could be a therapeutic strategy in clinical trials addressing this condition.

## 1 Introduction

Diabetic neuropathy, a frequent diabetes complication, afflicts nearly half of those with this enduring metabolic condition (1). This progressive degeneration of nerve fibers manifests in a spectrum of symptoms, from sensory disturbances like tingling and numbness in the limbs to severe outcomes such as ulcerations and limb amputations (2). Despite its significant impact on patient health, the underlying mechanisms of diabetic neuropathy are unclear, making it difficult to find effective treatments (3).

Emerging research implicated the perturbation of lipid metabolism as a pivotal factor in the onset of diabetic neuropathy, independent of glycemic status (4–7). In earlier research on diabetic neuropathy, the scope of investigation was confined to fundamental lipid profiles (7–9). However, the advent of sophisticated mass spectrometry techniques has revolutionized our capacity to detect and quantify extensive arrays of lipids, or lipidomes, in biological specimens. Lipidomic analysis holds the potential to pinpoint precise disease biomarkers and shed light on the underlying mechanisms of pathology (10, 11). This enhanced molecular resolution facilitates the creation of more tailored therapeutic strategies, promising a leap forward in the management of diabetic neuropathy.

Thus, we employed a two-sample Mendelian Randomization (MR) analysis, adhering to STROBE-MR guidelines, to investigate the causal links between 179 lipid species and the risk of diabetic neuropathy (12). MR study offers a robust method for exploring causal relationships between exposures and outcomes in epidemiology. Prior research leveraging MR methods has also emphasized some certain diseases associated with lipidomes and diabetic neuropathy (13–15). Using genetic variants as instrumental variables (IVs), MR overcomes confounding, providing more compelling evidence of causality than conventional observational studies (16).

## 2 Methods

### 2.1 Data sources

**Figure 1:**
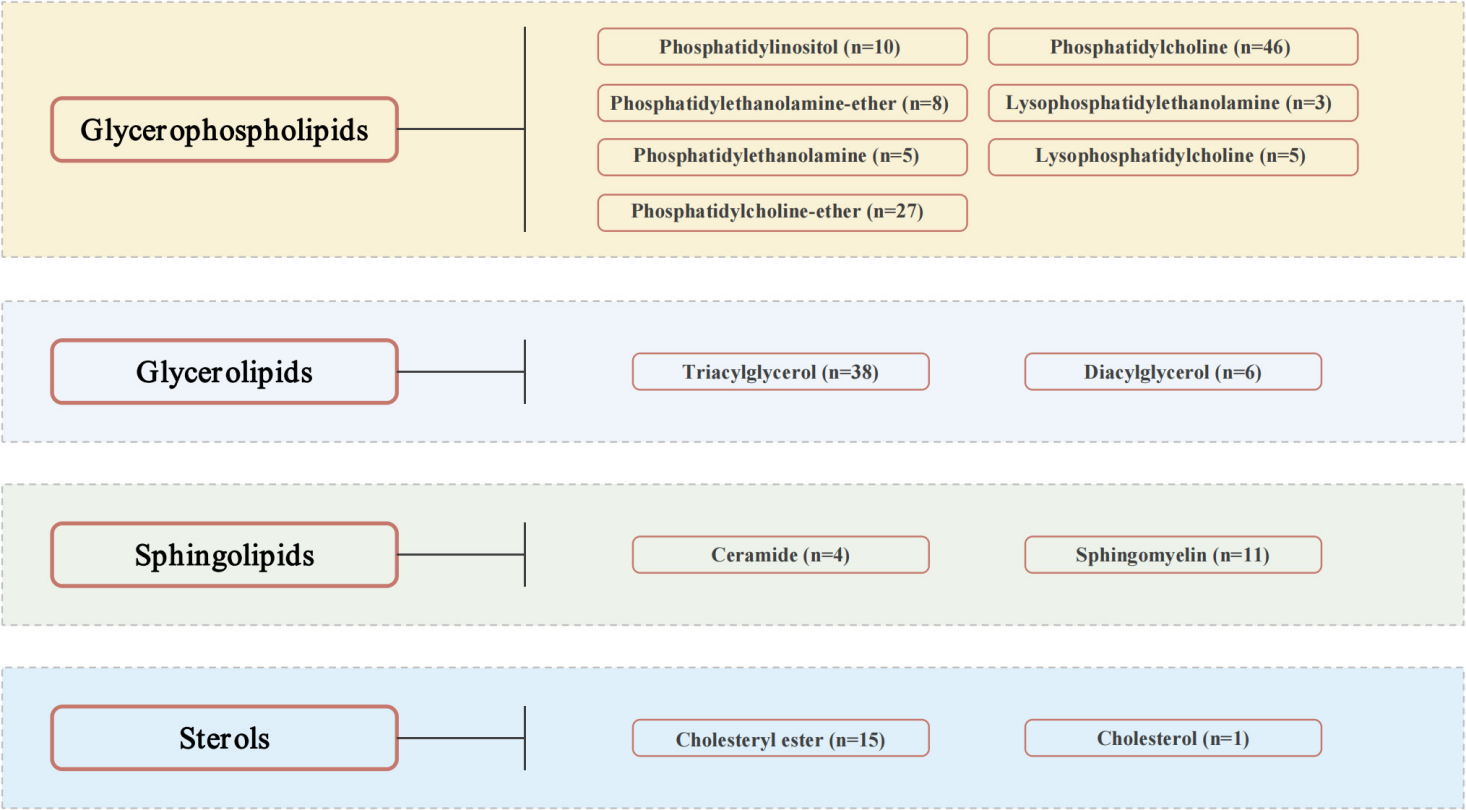
Lipid species.

A comprehensive GWAS dataset, featuring 179 lipid species across 13 classes and 4 major categories—glycerolipids, glycerophospholipids, sphingolipids, and sterols—is depicted in **Figure 1** (**Supplementary Data Sheet 1**) (17). These lipid species’ summary statistics are cataloged in the GWAS Catalog (https://www.ebi.ac.uk/gwas/) with accession numbers ranging from GCST90277238 to GCST90277416 (18). The dataset originates from the GeneRISK study, which includes 7,174 European participants (4,579 female and 2,595 male), recruited between 2015 and 2017, and aged 45–66. The primary goal of GeneRISK is to evaluate the effects of genetic risk information on cardiovascular disease (19). The original publication details the inclusion and exclusion criteria for participants, along with the population’s characteristics (19). Before blood sample collection for plasma, serum, and DNA extraction, participants observed a 10-hour fasting period. Collected biological and demographic data, alongside health, genetic, and lipidomic profiles, are stored at the THL Biobank (https://thl.fi/en/research-and-development/thl-biobank). Additionally, the summary-level statistics for diabetic neuropathy were retrieved from the R9 release of the FinnGen GWAS results, under the phenotypic classification “DM_NEUROPATHY”, with small overlap (<5%) with GWAS of lipid species, reducing the risk of bias (https://r9.finngen.fi) (20). The determination of diabetic neuropathy cases was anchored in the standardized International Classification of Diseases (ICD) coding system. The dataset included a total of 2,843 cases and 271,817 control individuals of European descent, with adjustments made for variables such as age, sex, genetic relatedness, genotyping batch, and the first 10 principal components. On the other hand, we also obtained the GWAS summary statistics for body mass index (BMI) and glycated hemoglobin (HbA1c) from the European cohorts, sourced respectively from the Genetic Investigation of ANthropometric Traits (GIANT) Consortium (https://portals.broadinstitute.org/collaboration/giant/index.php/GIANT_consortium) and the Meta-Analyses of Glucose and Insulin-related Traits Consortium (MAGIC) (https://magicinvestigators.org/) (21, 22).

### 2.2 Selection criteria for IVs

The MR analysis relied on three principal assumptions: (1) The IVs used in the study were significantly associated with lipidomes. (2) There was independence between the selected IVs and any confounding factors that could affect both lipidomes and diabetic neuropathy. (3) The IVs exerted an effect on diabetic neuropathy solely through their influence on lipidomes (23). To enable MR analysis across all lipid species, we adopted a significance threshold of *P* < 5e-6 for selecting genetic variants. This threshold is considered appropriate for MR studies when SNPs available for exposure are scarce (24, 25). To curtail the influence of correlated SNP associations, linkage disequilibrium (LD) scrutiny was performed using the European 1000 Genomes Project Phase 3 as a reference, with an r2 threshold of <0.001 and a clumping distance set at 10,000 kb. Palindromic SNPs were omitted to avoid inconsistencies in allelic interpretations that could skew causal inferences. SNPs with strong associations to the outcome were also discarded. The MR Steiger filter was applied to eliminate SNPs with an incorrect direction of effect. The IVs for lipid species were evaluated using the variance explained (R^2^) and the F-statistic, with those yielding an F-statistic below 10 being eliminated. The F-statistic is calculated using the formula R^2^(N-K-1)/[K(1-R^2^)], where R^2^ is the variance of the exposure explained by the IVs, N is the effective sample size, and K is the number of variants in the IV model. The PhenoScanner, an online resource, was utilized to identify and exclude SNPs linked with potential confounders (26). Finally, to evaluate the statistical power, we utilized the online tool available at https://shiny.cnsgenomics.com/mRnd/ (27).

### 2.3 MR analysis

The MR analysis was conducted using the R software (version 4.3.1), with specialized packages including “TwoSampleMR” (version 0.5.7), “MR-PRESSO” (version 1.0), and “MendelianRandomization” (version 0.9.0). A two-sample MR analysis was employed using five MR methods: inverse variance weighted (IVW), weighted median, simple mode, weighted mode, and MR-Egger regression. The primary method used was the fixed-effects IVW, which combined the Wald ratios from individual SNPs to provide a summary estimate. Additional methods served to validate the findings. Multiple testing was accounted for using False Discovery Rate (FDR) correction, with a significant association defined by an FDR of less than 0.05. Sensitivity analyses were performed to confirm the robustness of the MR results. Cochrane’s Q test assessed heterogeneity among IVs, with a *P* value below 0.05 indicating significant heterogeneity and necessitating the use of a random-effects IVW model instead of a fixed-effects model. The MR-Egger regression intercept was employed to detect potential horizontal pleiotropy, with significance set at a *P* value below 0.05. The MR-PRESSO global test was also used to assess horizontal pleiotropy, with outlier correction to mitigate its influence. The stability of the results was further examined using a leave-one-out strategy, where each SNP was sequentially excluded from the analysis, and the IVW method recalculated the effect. Finally, to explore the potential vertical pleiotropic pathways, we also performed multivariable MR (MVMR) analysis, including MVMR-IVW, MVMR-Egger, and MVMR-Median, to assess the direct causal impacts of these lipid species on diabetic neuropathy after adjusting for BMI and HbA1c levels. The parameter settings were consistent with univariable MR analysis.

## 3 Results

### 3.1 Selection of instrumental variables

After thorough quality control measures, we have pinpointed 2,722 SNPs to serve as IVs in examining the causal relationship between lipid species and diabetic neuropathy. Each of these SNPs has an F-statistic value exceeding 10, which suggests that the potential for bias due to weak instruments is minimal. Detailed information for each SNP, encompassing the effect allele, other allele, β value, standard error (SE), and *P* value, is provided in **Supplementary Data Sheet 2**.

### 3.2 Results of MR analysis between lipid species and diabetic neuropathy

**Figure 2:**
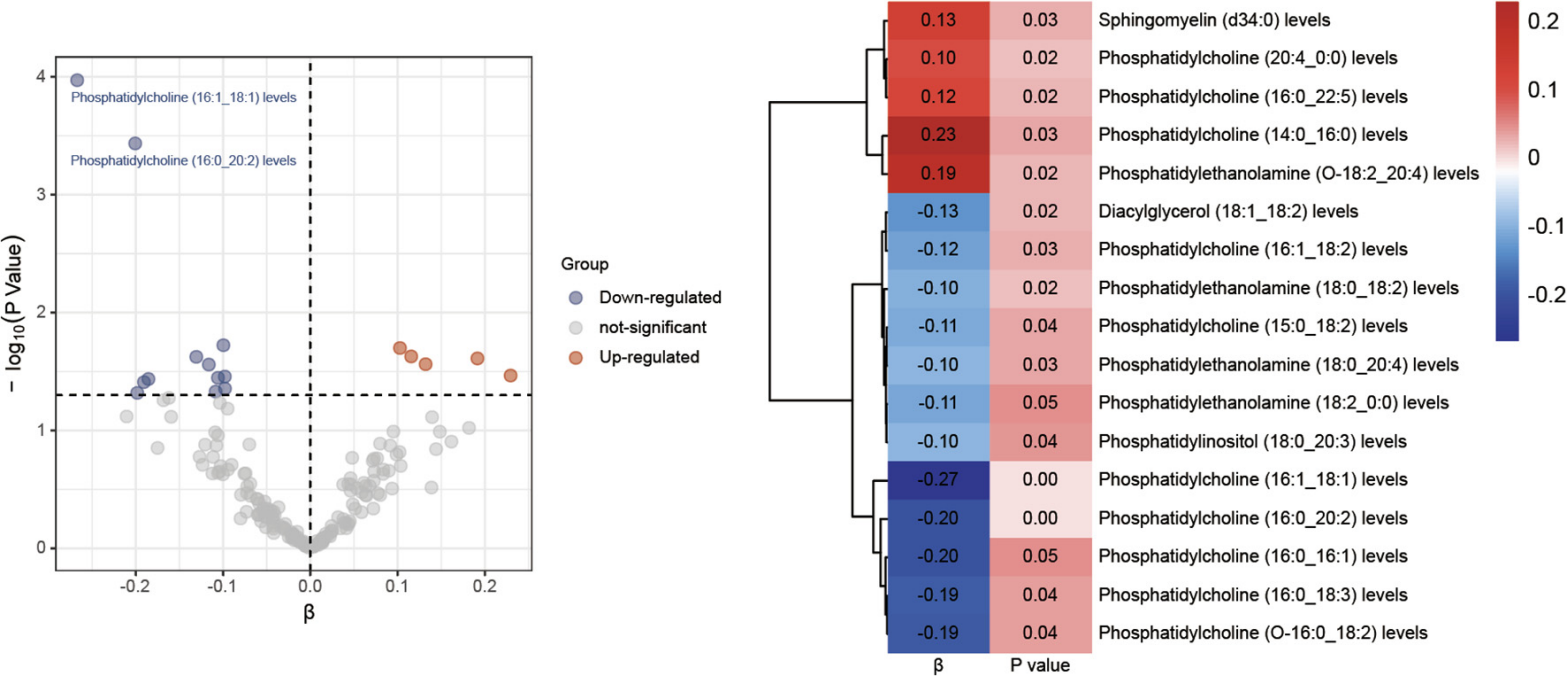
Volcano plots and heatmaps of differential lipid species.

A thorough MR analysis results of 179 lipid species and diabetic neuropathy was presented in **Supplementary Data Sheet 3**. Through the IVW analysis, we have identified 17 lipid species with potential causal relationship. Among these, 5 lipid species are identified as potential factors that may contribute to an elevated risk of diabetic neuropathy, whereas 12 lipid species seem to confer some protection against diabetic neuropathy, as illustrated in **Figure 2**. It is worth noting that phosphatidylcholine constitutes the majority.

**Figure 3:**
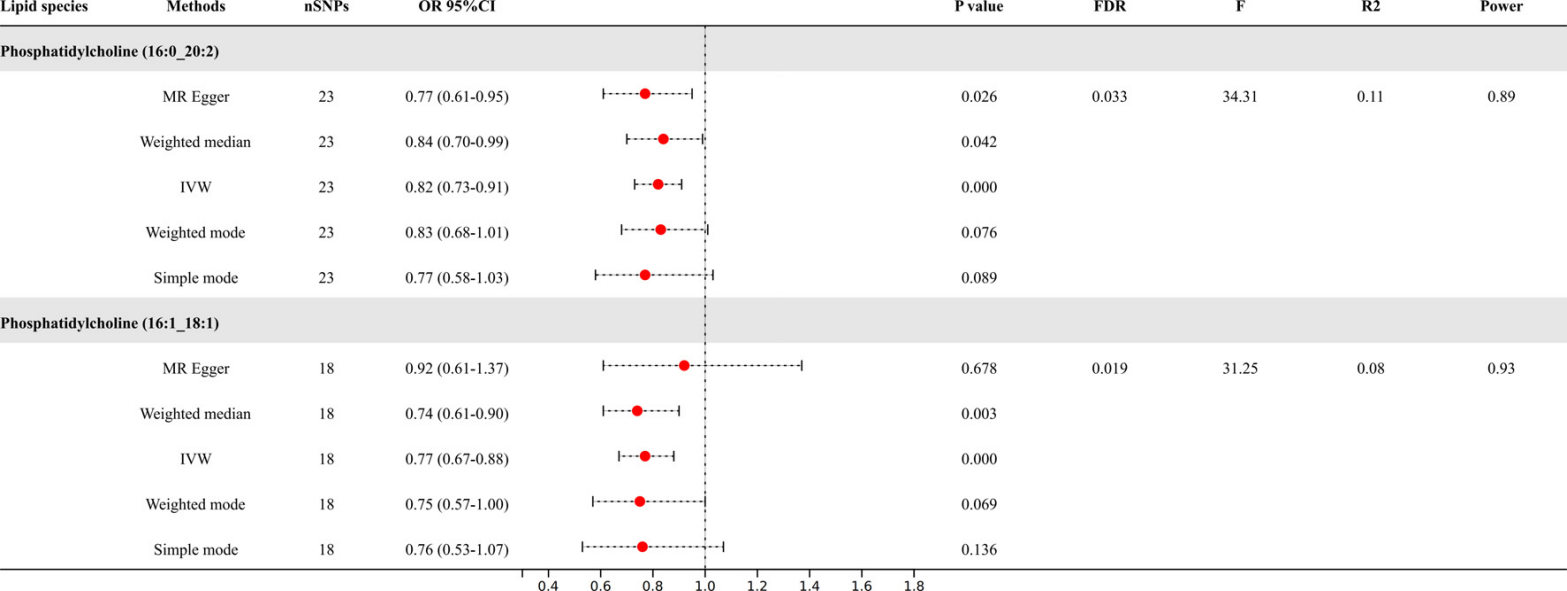
Causal relationships between two lipid species and diabetic neuropathy.

Following the application of FDR correction, two lipid species have been identified that show a significant causal link with the risk of diabetic neuropathy, as illustrated in **Figure 3**. Individuals with genetically higher levels of phosphatidylcholine (16:0_20:2) had a 18% lower risk of developing diabetic neuropathy (OR = 0.82, 95%CI: 0.73-0.91; *P* < 0.001, FDR = 0.033). Similarly, higher genetically inferred levels of phosphatidylcholine (16:1_18:1) were associated with a 23% reduced risk of diabetic neuropathy (OR = 0.77, 95%CI: 0.67-0.88; *P* < 0.001, FDR = 0.019). Additional analyses using methods such as MR-Egger, weighted median, simple mode, and weighted mode have also consistently indicated a trend in these causal associations. **Supplementary Data Sheet 4** presents scatterplots that clearly depict the causal connections between these lipid species and the risk of diabetic neuropathy.

### 3.3 Sensitivity analyses

Sensitivity analyses presented in **Table 1**, including the Cochrane’s Q test, showed no heterogeneity among the genetic variants associated with two lipid species in predicting diabetic neuropathy. The MR-Egger regression intercept, used to assess the risk of bias from unbalanced horizontal pleiotropy, suggested no significant impact on our findings concerning diabetic neuropathy. The robustness of our MR results was further confirmed by the MR-PRESSO test, reinforcing the reliability of our conclusions. **Supplementary Data Sheet 4** demonstrates that removing any single SNP in the leave-one-out analysis did not significantly change the MR estimates, affirming the IVW method as the preferred analytical strategy, given the lack of significant heterogeneity or unbalanced pleiotropy in explaining the variability in the risk of diabetic neuropathy (28–30).

**Table 1:** The results of sensitivity analyses.

Lipid species	Q_IVW_	*P*	Egger intercept	*P*	MR-PRESSO
Phosphatidylcholine (16:0_20:2)	17.961	0.708	0.012	0.495	0.779
Phosphatidylcholine (16:1_18:1)	19.513	0.300	-0.026	0.356	0.354

### 3.4 MVMR analysis

**Figure 4:**
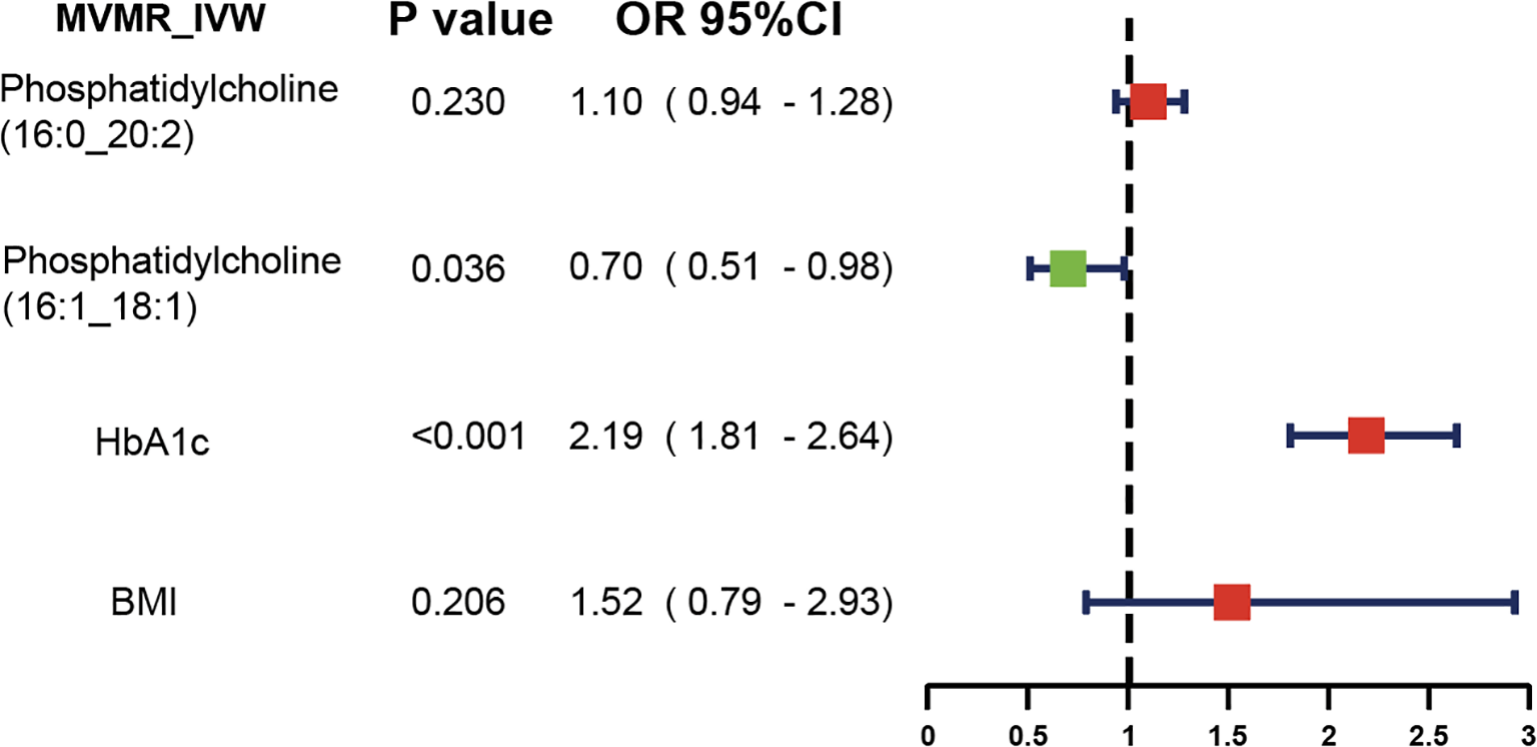
MVMR analysis results.

We further applied a MVMR analysis to assess the direct impacts of these two lipids on diabetic neuropathy (**Figure 4**). According to the MVMR-IVW findings, the effect of genetically predicted phosphatidylcholine (16:1_18:1) (OR = 0.70, 95% CI: 0.51-0.98; *P* = 0.036) on diabetic neuropathy remained significant after adjusting for BMI and HbA1c. The consistent direction and magnitude of results from the MVMR-Egger and MVMR-Median models further support the causal inference (**Supplementary Data Sheet 3**).

### 3.5 Results of MR analysis at genome-wide significance threshold (5e-8)

**Figure 5:**
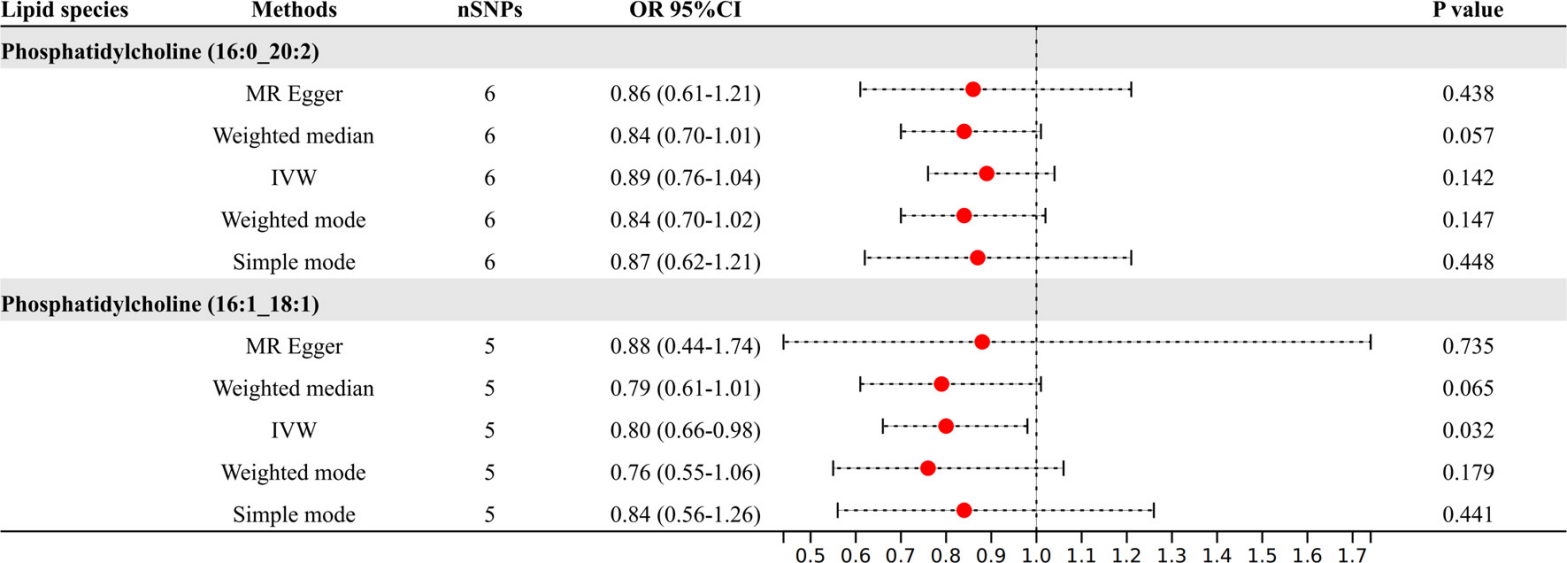
Results of MR analysis at genome-wide significance threshold (5e-8).

After refining our criteria based on a genome-wide significance threshold of *P <*5e-8, we selected IVs for these two lipid species (**Figure 5**). Phosphatidylcholine (16:0_20:2) had six SNPs as IVs; phosphatidylcholine (16:1_18:1) had five SNPs as IVs. Further IVW analysis indicated that the increased levels of phosphatidylcholine (16:1_18:1) remain as the protective factors for diabetic neuropathy (OR = 0.80, 95% CI: 0.66-0.98; *P* = 0.032).

## 4 Discussion

In our MR study, we explored the possible causal relationship between 179 lipid species and the risk of developing diabetic neuropathy. Our research identified 17 lipid species that appear to have potential causal links to the susceptibility to diabetic neuropathy.

Lipid metabolism plays a pivotal role in maintaining cellular membrane integrity, energy storage, signal transduction, and numerous other critical biological processes. The impact of dyslipidemia, which frequently co-occurs with diabetes, on the development of diabetic neuropathy is gaining recognition as a significant factor in its pathogenesis (4). Abnormal lipid metabolism heightens inflammation and oxidative stress which directly damage nerve fibers and contribute to neuropathy (31, 32). Lipid dysregulation can impair mitochondrial transport in sensory neurons and alter the generation of mitochondrial energy (33). Additionally, the integrity of Schwann cells, responsible for the myelination of peripheral nerves, relies on proper lipid metabolism (34). Several clinical studies have demonstrated significant alterations in the plasma lipidome of diabetic patients, which may be associated with the onset and progression of neuropathy. For instance, changes in specific lipid subclasses, such as phospholipids and sphingolipids, have been correlated with the severity of nerve damage (7, 35). Through the MR studies, we have enhanced the genetic evidence, identifying novel lipid species potentially linked to the susceptibility of diabetic neuropathy, along with their possible pathogenic and protective impacts. After adjusting for multiple testing, we have pinpointed two lipid species that demonstrate significant causal relationships. Phosphatidylcholine (16:0_20:2) and phosphatidylcholine (16:1_18:1) exhibit protective effects against diabetic neuropathy. Phosphatidylcholine is crucial for maintaining cell structure and function, regulating metabolism, facilitating signal transduction, and transporting lipids (36). Research indicates that a high intake of choline, particularly in the form of phosphatidylcholine, is associated with a reduced risk of type 2 diabetes (37). On the other hand, inhibiting the remodeling of phosphatidylcholine in adipose tissue can enhance insulin sensitivity (38). A prior investigation into the lipidomic profile associated with type 2 diabetic neuropathy also revealed that subjects with neuropathy exhibited a reduction in phosphatidylcholine levels, regardless of the fatty acid chain length and degree of saturation (7). Among participants experiencing peripheral neuropathy, alterations in phosphatidylcholine levels are also related to impaired mitochondrial β-oxidation (7). However, it is important to note that the potential physiological mechanisms by which these lipid species combat diabetic neuropathy are not yet clear and require further exploration. Additionally, the development of targeted therapeutic agents remains an area for future research.

This study’s conclusions come with certain limitations that could affect their interpretation. Firstly, the SNPs utilized fell short of the standard genome-wide significance threshold of 5e-8; instead, a more lenient criterion of 5e-6 was applied for IVs selection. Secondly, the study primarily included participants of European descent, reducing population variability, yet it underscores the need to confirm the MR findings in various ethnic populations for wider applicability. Thirdly, our data were sourced from registries, which may contain inconsistencies and errors, particularly in the classification of diabetic neuropathy. The FinnGen database does not offer a detailed classification of diabetic neuropathy, such as distinguishing painful diabetic neuropathy. Lastly, while MR analysis is valuable for inferring causal relationships, validating these findings through rigorous randomized controlled trials is essential.

## 5 Conclusion

This study revealed associations between specific lipid species and the risk of diabetic neuropathy, illuminating the potential of lipid-targeted therapies in clinical trials aimed at combating diabetic neuropathy.

## Data Availability

The original contributions presented in the study are included in the article/**Supplementary Material**, further inquiries can be directed to the corresponding author/s.

## Supplementary material

Supplementary Data Sheet 1 Lipid species.

Supplementary Data Sheet 2 The detailed characteristics of IVs.

Supplementary Data Sheet 3 MR analysis results.

Supplementary Data Sheet 4 Scatter plots and leave-one-out method results.
