# Supplementary figures and images for: Causal relationships between plasma lipidome and diabetic neuropathy: a Mendelian randomization study
Source: Front Endocrinol (Lausanne). 2025 Jan 15;15:1398691. doi: 10.3389/fendo.2024.1398691 (PMC11774734; doi:10.3389/fendo.2024.1398691)

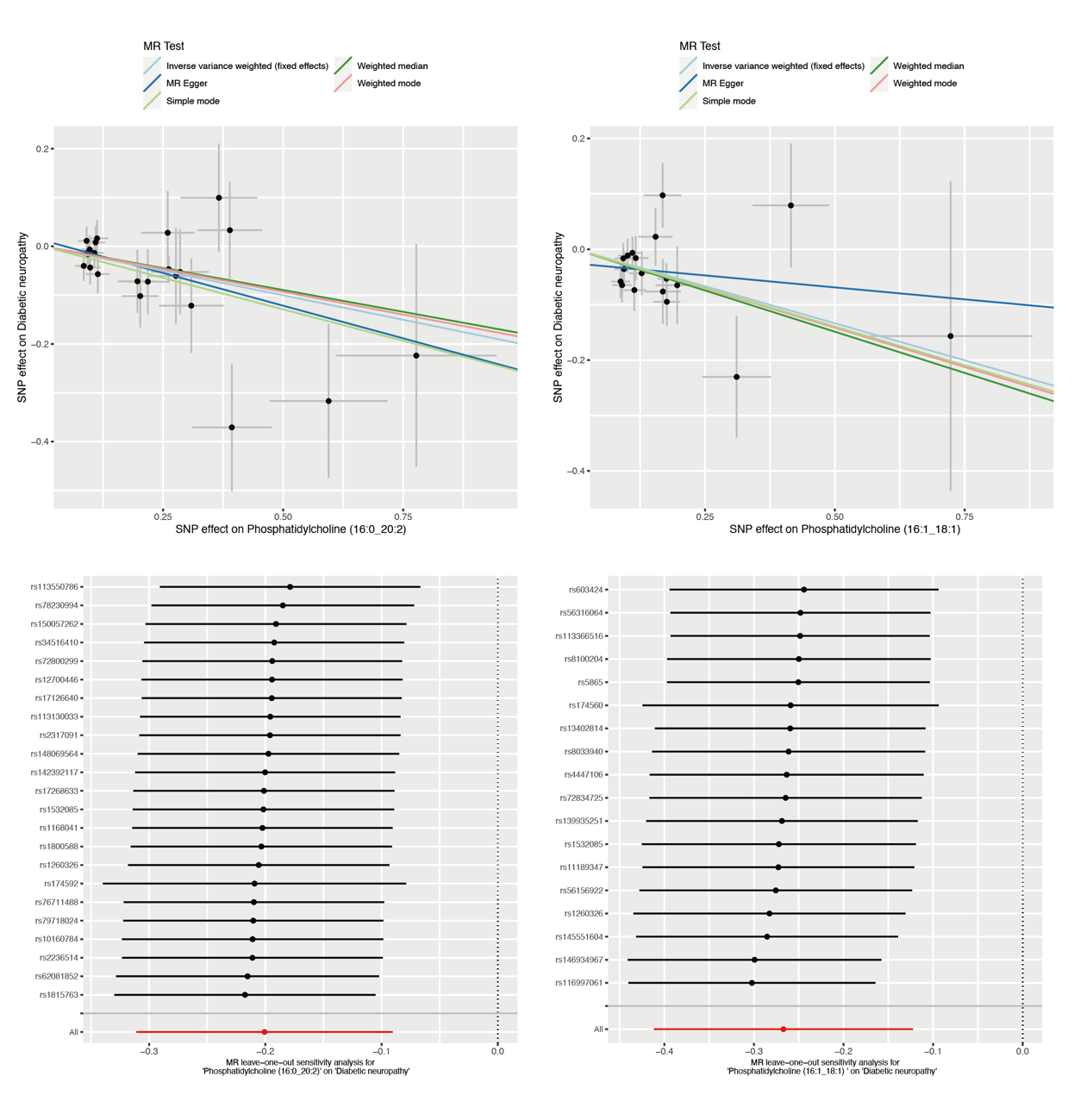

Supplement: Supplementary Data Sheet 4 — Scatter plots and leave-one-out method results. [file DataSheet4.docx]
